# Women’s socioeconomic status and attitudes toward intimate partner violence in Eswatini: A multilevel analysis

**DOI:** 10.1371/journal.pone.0294160

**Published:** 2023-11-13

**Authors:** Garikayi B. Chemhaka, Stanzia Moyo, Maswati S. Simelane, Clifford Odimegwu

**Affiliations:** 1 Department of Statistics and Demography, Faculty of Social Sciences, University of Eswatini, Kwaluseni, Manzini, Eswatini; 2 Department of Demography Settlement and Development, Faculty of Social and Behavioural Sciences, University of Zimbabwe, Harare, Zimbabwe; 3 Demography and Population Studies Programme, Schools of Social Sciences and Public Health, University of the Witwatersrand, Braamfontein, Johannesburg, South Africa; Northeastern University, UNITED STATES

## Abstract

**Introduction:**

Attitudes supportive of spousal violence retards developmental efforts worldwide, and in particular in patriarchal African settings. It is important to curb this behavior by designing preventative evidence-based policies. This study examines the acceptance of intimate partner violence among women residing in Eswatini and determines whether attitudes supportive of intimate partner violence are associated with women’s low socioeconomic status both at the individual- and community-level.

**Methods:**

Cross-sectional secondary data from two Eswatini Multiple Indicator Cluster Surveys (MICS) conducted in 2010 and 2014 with representative samples of 4,686 and 4,761 women, respectively were analyzed using descriptive statistics and multilevel (random effect) logistic regressions.

**Results:**

Overall, the prevalence of acceptance of intimate partner violence declined significantly between 2010 and 2014 in Eswatini (29.0% vs. 19.8%, p<0.001). In both surveys, socioeconomic factors associated with women’s supportive attitudes toward intimate partner violence were educational level, marital structure, and community socioeconomic disadvantage. Overall, primary or lower educational attainment, single/unmarried relationships, and women living in a community with a high socioeconomic disadvantage were key factors associated with supportive attitudes toward intimate partner violence.

**Conclusions:**

Secondary or higher education for individual women and a high proportion of women in the community with low socioeconomic disadvantage are important socioeconomic predictors of reducing women’s supportive attitudes toward intimate partner violence. Therefore, further gains in non-supportive attitudes toward acceptance of intimate partner violence could be achieved through efforts and intervention in the education of individual women and improving women’s socioeconomic status in the community.

## Introduction

Violence against women has continued to draw global attention to women’s population, health, and rights issues [1–5]. The Sustainable Development Goals (SDGs) of the United Nations and other international development initiatives have also given prominence to violence against women. For developing countries, promoting peace (SDG17), good health (SDG3), high-quality education (SDG4), gender equality (SDG5), and decreasing inequalities (SDG10) targets continue to be priorities, especially for women [6].

Despite the existence of the aforementioned policies, objectives, and strategies for development, it should be underlined that violence against women continues to be a problem for the least developed countries, which are the most affected [7, 8]. According to a 2018 World Health Organization report, 27% of women worldwide reported they had ever experienced intimate partner violence, with a third of those cases occurring in Southeast Asia and Africa [7]. In the same report, intimate partner violence in Africa ranged from 16% in Comoros to 47% in the Democratic Republic of Congo. According to a survey done in six East African countries, the prevalence of intimate partner violence in the 12 months before the survey ranged from 28% to 39% [9].

According to recent literature, although intimate partner violence violates human rights and has significant social and health consequences, and ultimately mortality in all societies [5, 10–14], it is nonetheless tolerated to diverse degrees in several countries and regions. For instance, in the European Union, 84% of people perceived that intimate partner violence against women was unacceptable and should be criminalized [15]. In 49 low- and middle-income nations, women were more likely than men to justify intimate partner violence (41% vs. 32% overall) [16]. In comparison to their peers in Sub-Saharan Africa (45%) and South/South-East Asia (53%), women in Latin America and the Caribbean (12%), Central/West Asia (24%) and Europe (24%) had less pervasive views that justify violence. In Sub-Saharan Africa, attitudinal acceptance of violence against women among women ranged from 13% in Malawi to 92% in Guinea [16]. According to a study of South Asian nations, there are varying degrees of women’s acceptance of intimate partner violence. Examples include when a woman leaves her husband without telling him, which ranges from 28% in Nepal to 82% in Afghanistan; neglects her children, which ranges from 30% in Pakistan Punjab to 65% Afghanistan; argues with her husband, which ranges from 19% Nepal to 80% Afghanistan; refuses to have sex with her husband, which ranges from 3% in Nepal to 54% Afghanistan; and burns food, which ranges from 5% Nepal to 35% in Afghanistan and Pakistan Sindh [4].

Several theories have been postulated to explain the several factors underlying the prevalence of violence including social learning, patriarchal, social-ecological, and resources [1, 3, 11, 17–19]. For instance, the social learning theory assumes that experiencing household violence, particularly during childhood, either as a victim or a witness, leads to subsequent intimate partner violence, through perpetration or acceptance of violent norms or attitudes. According to the patriarchal view, women are subjugated because of the social structures and practices that men use to assert their authority and power in patriarchal societies. Interestingly, a recent study on how gender influences attitudes toward acceptance of intimate partner violence found that women are more likely than men to approve of using violence [19]. More so, there is a lack of empirical study on how women adopt attitudes tolerating violence [19].

Notwithstanding the importance of social learning and patriarchal theories in the violence discourse, it should be noted that the social-ecological and resource theories also provide key assumptions underlying the attitudinal acceptance of violence among women. For instance, the social-ecological model acknowledges that individual factors (biological and personal history) influence exposure to violence, and it also places emphasis on the interplay of community and structural factors, especially concerning the acceptability of violence [3, 18, 20]. Community-level factors refer to the extent to which violence is tolerated in contexts in which social relationships are embedded [3, 20]. Heise adds that social factors such as the unequal distribution of power between women and men and cultural and social norms that shape gender roles are among these. Therefore, it is assumed that intimate partner violence occurs more often in societies where men have economic and decision-making powers in the household, where it is more difficult for women to get a divorce, and where adults routinely resort to violence to resolve their conflicts [3]. According to Heise [3], the structural factors include rigid gender roles, acceptance of interpersonal violence, acceptance of physical chastisement, and masculinity associated with aggression and dominance.

The social-ecological model resonates with the feminist perspective, which asserts that the patriarchal domination of women through “wife abuse” is a holdover from the long cultural history of legally sanctioned male abuse, control, and outright ownership of women [21]. According to resource theory, individual low socioeconomic status among women is associated with the acceptance of violence [1, 11, 22]. Due to their dependence on their spouses, women with limited resources are more prone to abuse. On the other hand, unexpectedly, women with more resources may experience greater abuse due to the stress that comes from changing their status as subservient women, or “status inconsistencies” [11]. Both theories operate at the micro (individual/couple) and macro (community) levels, although studies on intimate partner violence have mostly ignored the latter analytical level [11, 23].

Therefore, adapting social-ecological, and resource theories, our study hypothesizes that women with limited resources in terms of wealth, and education, as well as those who live in communities with socioeconomic disadvantage, are more exposed to the risk of accepting intimate partner violence. In addition, older women, in polygynous (unequal) relationships, and living in disadvantaged communities such as rural areas are more likely to accept intimate partner violence. However, due to the complexity of these factor interactions and effects in the literature [24], we did not evaluate them in our study design. Evidence has shown that one of the most important predictors of intimate partner violence is the attitude towards intimate partner violence [23]. Therefore, promoting and comprehending women’s aversion to tolerating behaviors that support their abusers is important.

Women’s educational level has been negatively associated with justifying acceptance of intimate partner violence [2, 11, 12, 16, 25, 26]. A study of 49 low- and middle-income countries showed along with poverty, education had the strongest protective effect on women’s societal acceptance of intimate partner violence [16]. According to the study, women in societies with higher levels of female literacy had a lower likelihood of justifying intimate partner violence. Household wealth is a strong predictor of women’s acceptance of intimate partner violence [12, 24–26]. In general, poverty or socioeconomic disadvantage is linked with a higher risk of justifying intimate partner violence, while employment and wealth tend to reduce the risk of women justifying this violence. Age [2, 12, 24, 26], marital structure [12, 25–27], urban-rural residence, and region of residence [2, 12, 26, 28] are sociodemographic factors that are significant predictors of women’s acceptance of intimate partner violence.

Gender is an underlying factor in the acceptance of intimate partner violence against women [2, 16, 29]. For instance, in countries with higher societal acceptance of intimate partner violence, women were more likely than men to justify intimate partner violence. For example, in Guinea and Niger, women were almost 6 and 4 times more likely than men to justify intimate partner violence, respectively [16]. In a patriarchal society, women are frequently socialized to accept abuse as a kind of conflict resolution or as a form of punishment for disobedience [16, 24, 27, 29]. In Eswatini and elsewhere in Africa, the acceptability of intimate partner abuse is quite common among women, especially among those underprivileged [26, 30–32]. Other studies, however, reveal lower levels of acceptability of intimate partner violence among women than men [24].

It should be noted that there is a gray area in research, particularly in assessing and comparing the prevalence and predictors of attitudinal acceptance of intimate partner violence among women in Eswatini between 2010 and 2014. This is not to diminish the significance of the aforementioned literature about the discourse on intimate partner violence at the global and regional levels. Yet available literature shows that Eswatini is a patriarchal society that perceives women as minors as entrenched in traditional customs such as paying ‘lobola’ bridewealth [31, 33]. Women are vulnerable to rape, assault, or other forms of violence in Eswatini [34]. For example, childhood sexual violence was reported among young women, in particular, those not attending school [35]. Intimate partner violence against women has been reported to increase after HIV infection [36]. Girls were reported to have had high emotional abuse associated with an economic disadvantage compared to boys [37].

Eswatini is a signatory to international and regional policies aimed at curbing gender-based violence. In 2018, the Sexual Offences and Domestic Violence Act, No. 15 was enacted into law. The aim of the law included *inter alia*: to promote the realization of the rights of women and girls in the Kingdom of Eswatini, and to make all forms of sexual abuse or exploitation a crime [38].

There is a dearth of research on supportive attitudes toward intimate partner violence among women and communities in Eswatini that uses nationally representative data. While intimate partner violence against women is a cause of concern, there are knowledge gaps that exist about women victims justifying intimate partner violence against them. Women often encounter additional barriers and cultural/social norms that restrict their choices in power dynamics [39]. In rural communities, patriarchal cultural beliefs and practices are highly respected and upheld, particularly through the leadership of Chiefs and other Swazi traditional authorities [31, 38, 40]. According to an earlier study, about 2 out of 5 Swazi women and men justified intimate partner violence against women [14]. Research conducted in other contexts has revealed that in addition to individual socioeconomic disadvantage, neighborhood or community socioeconomic disadvantage also increases the likelihood of intimate partner violence against women [22, 41, 42], although one study found no evidence of this relationship [20]. To our knowledge, there hasn’t been much research on the possible impacts of community socioeconomic disadvantage on women’s risk of accepting or tolerating this kind of violence, despite the literature’s conclusions that it is a risk factor for intimate partner violence.

Our current study adapts the social-ecological and resource theories in a multilevel framework to assess the socioeconomic status predictors underlying the acceptability of intimate partner violence among women in Eswatini between 2010 and 2014. Examining attitudes justifying intimate partner violence against women is of sheer importance since affirming or rejecting such attitudes nourishes the ecosystem in which violence takes place [24, 29].

## Materials and methods

### Participants

The study utilizes data from the 2010 and 2014 Eswatini Multiple Indicator Cluster Surveys (EMICS) for individual women collected by the Central Statistics Office. Multiple Cluster Survey (MICS) is a UNICEF-funded initiative in many developing countries to collect cross-sectional, nationally representative household sample surveys, containing various demographic and socioeconomic characteristics, on the situation of children, men, women, and the household.

The 2007 Eswatini census sampling frame was utilized in the MICS surveys conducted in 2010 and 2014. A two-stage sampling procedure was used; households were selected from enumeration areas (EAs) or clusters using a systematic sampling procedure stratified by urban-rural and four regions of residence (Mbabane, Manzini, Lubombo, and Shiselweni), with a probability proportionate to the size of each geographic area. In 2010, a sample target of 365 EAs with 5,475 households was listed nationally. Of these, 5,074 were selected, and 4,834 were interviewed, for a 95.3% response rate. In these households, a total of 4,688 women aged 15–49 were interviewed and a 94.6% response was achieved. In 2014 MICS, a total of 5,211 households in 347 EAs were listed, and 4,981 and 4,865 were selected and interviewed, respectively yielding a 97.7% response rate. In these households, a total of 4,762 women aged 15–49 were interviewed and the response rate was 95.2%. The sample with non-missing data was extracted for all women aged 15–49 years in the women file; 4,686 (2010) and 4,761 (2014). Details of these MICS surveys are described in detail elsewhere [43, 44].

### Measures

The dependent variable of interest is the acceptance or justification of intimate partner violence among women and is based on a set of five questions that are commonly asked in MICS. Women were asked whether or not they agree a husband is justified in beating his wife if she did any of the following: (1) goes out without telling him, (2) neglects the children, (3) argues with him, (4) refused to have sex, or (5) burned the food. These five questions were used to construct a binary variable for supportive attitudes toward intimate partner violence against women. Any affirmative answer to one or more of the questions was identified as accepting intimate partner violence and otherwise for rejecting all the five given scenarios.

The explanatory socioeconomic variables constituted both individual- and community-level characteristics and were derived from the literature and dataset. Individual-level variables are as follows: household wealth (poor, middle, and rich), education (none, primary, and secondary or higher), marital structure (unmarried, married monogamous, married polygynous, or formerly married), and age group (15–19, 20–29, 30–39, and 40–49), and community variables include; place of residence (rural, urban), region of residence (Hhohho, Manzini, Shiselweni, Lubombo), and community socioeconomic disadvantage (low, high). The community variable is the proportion of women in the community with social and economic status disadvantages. A community socioeconomic status variable was derived as in previous studies [45, 46], which incorporated social status variables to assess a lack of resources dimension. The percentage of women in the community with a socioeconomic disadvantage was derived by aggregating women with primary or less education and those with poor household wealth. Household wealth is an index measure in the dataset that is computed using the principal component analysis method from groupings of household assets and housing conditions [47].

### Ethical consideration

The UNICEF team granted permission for the access and use of the Eswatini MICS dataset from http://mics.unicef.org/surveys. The data are publicly available on request on the UNICEF MICS data repository. They are anonymized and participants cannot be identified. The authors had no special access privileges to the data and other researchers will be able to access it in the same manner as the authors.

### Statistical analysis

Stata 15.0 was used for data analysis and data were weighted using complex survey commands. Descriptive statistics for all characteristics in the study sample were presented using frequencies and percentages. The prevalence of supportive attitudes toward intimate partner violence is reported by sample characteristics. The association between women’s socioeconomic status and acceptance of intimate partner violence was examined using the chi-square test. All variables selected for the study were retained to allow comparisons between the 2010 and 2014 surveys. Adjusted odds ratios were calculated using multivariate multilevel logistic regressions to show the net associations between independent variables and acceptance of intimate partner violence. The equation for multilevel logistic regression is given in the form [48]:

$$logit\left(\pi_{ij}\right)=\beta_{0}+\beta_{i}x_{ij}+\zeta_{j}$$

Where *π*_*ij*_ is the proportion of women who are supportive of intimate partner violence, *β*_0_ is the intercept, *β*_*i*_ represents coefficients of the individual and community level factors, **x**_*ij*_ are the individual and community level explanatory variables, and *ζ*_*j*_ represents random errors at cluster or community level.

The results are reported at a 95% confidence interval (95% CI) and a p-value less than 0.05 was considered statistically significant. All socioeconomic characteristics fitted were not collinear. Multicollinearity was tested using the variance inflation factor (VIF) with a value greater than 10 indicating severe multicollinearity. The Wald test was used to assess the goodness fit of the final model. The Akaike information criterion (AIC) was used to test the models.

Since individual women are nested in clusters and simultaneously individual and community-level socioeconomic characteristics are examined, a random intercept multilevel (two-level) logistic regression was adopted. A four-step random intercept logistic modeling approach was adopted; the empty model (model 1), which had no explanatory variables, showed there is a significant variation of acceptance of intimate partner violence among communities (or clusters), hence a multilevel approach was deemed appropriate. Individual- and community-level variables were put in models 2 and 3, respectively to determine their independent effects on the outcome. The final model (model 4) combines both individual and community characteristics, as well as community random effects, and was used for comparison of results across both 2010 and 2014 surveys. While fixed effects (measures of association) are measured by odds ratios, the random effects (measures of variation) were measured using the intra-cluster correlation coefficient (ICC), median odds ratio (MOR), and the proportion change in variance (PCV), capturing heterogeneity in acceptance of intimate partner violence among women across communities [49]. The MOR is an adjusted between-area variance measure to use the same scale as the estimates of the odds ratios at the individual level. High MOR values (2 or higher) suggest a greater between-area variation while values close to 1 suggest little change between areas [49]. As a rule of thumb, the higher the variance value the greater the heterogeneity between communities or cluster areas.

## Results

Table 1 shows the distribution of the study sample by survey years. Of the total sample of women included in the analysis in 2010 (4,686) and 2014 (4,761), there was slightly one-third (32.81%) in 2010 and (33.39%) in 2014 poor households. In both survey years, a majority of women were young (aged 20–29 years or less), unmarried, had primary or above education, resided in rural areas, and lived in the Manzini region.

**Table 1:** Sample distribution of women aged 15–49 in Eswatini, 2010–2014.

Characteristics	MICS 2010		MICS 2014	
	N	%	N	%
**Household wealth**				
Poor	1,435	32.81	1,902	33.39
Middle	906	19.85	1,042	19.76
Rich	2,345	47.34	1,817	46.85
**Education**				
None	1,453	32.25	1,372	26.92
Primary	1,564	33.95	1,721	35.64
Secondary or higher	1,669	33.79	1,668	37.44
**Marital structure**				
Unmarried	2,392	50.38	2,410	49.96
Monogamous	1,289	28.35	1,382	28.67
Polygynous	193	4.247	199	3.867
Formerly married	812	17.03	770	17.5
**Age group**				
15–19	1,078	23.42	1,112	21.79
20–29	1,765	37.33	1,639	35.36
30–39	1,066	22.42	1,164	25.37
40–49	777	16.83	846	17.49
**Place of residence**				
Rural	2,930	71.14	3,685	67.66
Urban	1,756	28.86	1,076	32.34
**Region of residence**				
Hhohho	1,211	27.43	1,260	24.54
Manzini	1,309	32.34	1,308	40.39
Shiselweni	1,142	22.03	1,223	16.78
Lubombo	1,024	18.21	970	18.28
**Community socioeconomic disadvantage**				
Low	2,389	47.53	2,384	59.55
High	2,297	52.47	2,377	40.45
**Total**	**4,686**	**100**	**4,761**	**100**

### Prevalence in attitudes toward intimate partner violence

**Fig 1:**
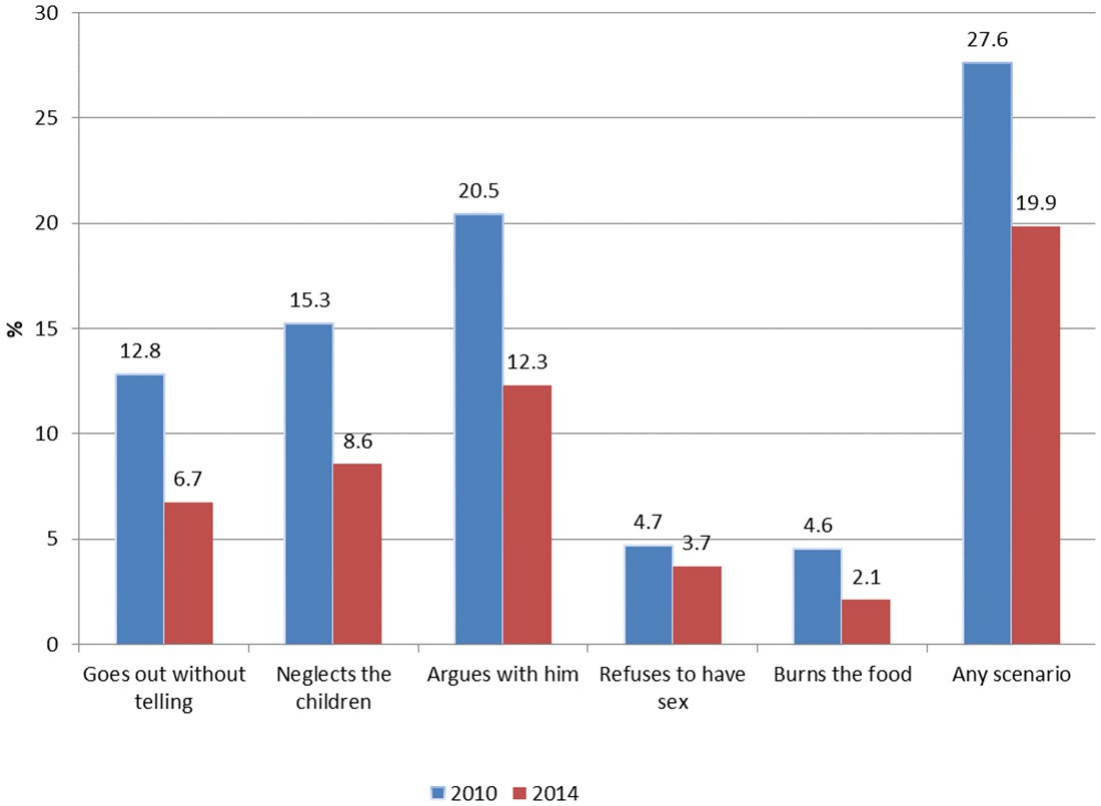
Prevalence of acceptance of intimate partner violence among women aged 15–49, 2010 and 2014.

Fig 1 shows a decline in the supportive attitudes toward intimate partner violence among Eswatini women between 2010 and 2014. Overall, the prevalence of attitudinal acceptance of intimate partner violence reduced by 28.1% between 2010 and 2014 (27.6% vs. 19.9%), p<0.001. This reduction was observed among all the various attitudinal intimate partner violence questions: burning food (54.3%), going out without telling their partner (47.7%), neglecting the children (43.8%), arguing with him (40%) and refusing sexual intercourse (21.3%).

### Bivariate analysis

Table 2 shows the results of the overall prevalence of acceptance of intimate partner violence among the women by explanatory variables in each survey year, with the corresponding p values based on the Chi-square test. The poor households had a significantly higher proportion of women with acceptance of intimate partner violence versus the poorest in 2010 (41.65% vs. 18.26%), p<0.001. In both survey years, the proportion of women’s acceptance of intimate partner violence significantly differed by women’s educational status (p<0.001). In 2010, acceptance of intimate partner violence was significantly different by marital structure: unmarried (33.18%), monogamous (21.55%), polygynous (31.63%), and formerly married (28.92%) (p<0.001). There was no significant difference in acceptance of intimate partner violence in 2014. Women’s acceptance of intimate partner violence was also significantly different by age of women, place of residence, and region of residence (all p < 0.001 in both years). In both survey years, the majority of the women who accepted intimate partner violence were residents in communities with high socioeconomic disadvantage versus low socioeconomic disadvantage (38.06% vs. 17.87% in 2010 and 26.9% vs. 17.59%), (p<0.001).

**Table 2:** Socioeconomic status and attitudes of acceptance of intimate partner violence by women aged 15–49 in Eswatini, 2010–2014.

Characteristics	MICS 2010			MICS 2014		
	N	%(Yes)	p-value	N	%(Yes)	p-value
**Household wealth** ***			<0.001			<0.001
Poor	1,435	40.64		1,902	27.73	
Middle	906	30.40		1,042	23.74	
Rich	2,345	17.46		1,817	12.64	
**Education** ***			<0.001			<0.001
None	1,453	38.64		1,372	26.80	
Primary	1,564	28.43		1,721	23.12	
Secondary or higher	1,669	16.32		1,668	11.80	
**Marital structure** ***			<0.001			<0.001
Unmarried	2,392	32.02		2,410	24.15	
Monogamous	1,289	20.04		1,382	13.07	
Polygynous	193	31.57		199	19.60	
Formerly married	812	26.31		770	18.88	
**Age group** ***			<0.001			<0.001
15–19	1,078	42.27		1,112	32.29	
20–29	1,765	26.47		1,639	19.99	
30–39	1,066	19.9		1,164	11.63	
40–49	777	20.16		846	16.13	
**Place of residence** ***			<0.001			<0.001
Rural	2,930	33.25		3,685	23.29	
Urban	1,756	13.80		1,076	12.74	
**Region of residence** ***			<0.001			<0.001
Hhohho	1,211	30.11		1,260	17.06	
Manzini	1,309	18.28		1,308	17.05	
Shiselweni	1,142	33.91		1,223	24.01	
Lubombo	1,024	32.92		970	26.10	
**Community socioeconomic disadvantage** ***			<0.001			<0.001
Low	2,389	17.44		2,381	14.94	
High	2,297	36.86		2,380	27.13	
**Total**	**4,686**	**27.6**		**4,761**	**19.9**	

***P<0.001,

**P<0.01,

*P<0.05

### Multilevel analysis

The measures of association (fixed effects) and variation (random effects) results are shown in Tables 3 and 4, respectively for both 2010 and 2014 Eswatini MICS surveys. For brevity purposes in this study, a fixed effects final model (model 4) that combines both individual and community factors in multilevel analysis is interpreted with emphasis. In 2010, after adjusting for all other factors, women aged 15 to 19 years, poor, uneducated, in polygynous relationships, living in rural areas or the Hhohho region, and living in communities with a high socioeconomic disadvantage were more likely to accept intimate partner violence. In 2014, women who were uneducated, unmarried, and from a community with a high socioeconomic disadvantage were more likely to justify intimate partner violence. After adjusting for individual and community level factors, in 2010, women from poor households (AOR = 1.75, 95% CI: 1.33, 2.29) and average households (AOR = 1.40, 95% CI: 1.07, 1.83) were more likely to accept intimate partner violence compared to women from rich households. In 2014, there was no significant association between household wealth and women’s acceptance of intimate partner violence. In 2010, the results showed that women with no education and primary education were significantly more likely to accept intimate partner violence (AOR = 2.34, 95% CI: 1.83, 2.97, and AOR = 1.48, 95% CI: 1.16, 1.89; respectively) compared to those with secondary or higher education. Similarly, in 2014, women with no education and primary education were more likely to accept intimate partner violence (AOR = 3.66, 95% CI: 1.57, 8.53, and AOR = 3.42, 95% CI: 1.29, 9.05; respectively). Thus, women’s education was protective against justifying intimate partner violence.

**Table 3:** Factors associated with attitudes of acceptance of intimate partner violence by women aged 15–49 in Eswatini, 2010–2014. Results from the final model of two-level logistic regression model.

Fixed effects	AOR (95% CI)	AOR (95% CI)
	MICS 2010	MICS 2014
**Household wealth**		
Poor	1.75(1.33–2.29)***	1.12(0.76–1.65)
Middle	1.40(1.07–1.83)*	1.11(0.70–1.76)
Rich (ref.)	1.00	1.00
**Education**		
None	2.34(1.83–2.97)***	3.66(1.57–8.53)**
Primary	1.48(1.16–1.89)**	3.42(1.29–9.05)*
Secondary or higher (ref.)	1.00	1.00
**Marital structure**		
Unmarried	1.44(1.09–1.90)**	3.21(1.00–10.32)*
Formerly married	1.55(1.16–2.07)**	2.70(0.89–8.17)
Polygynous	1.78(1.16–2.74)**	1.23(0.68–2.22)
Monogamous (ref.)	1.00	1.00
**Age group**		
15–19	2.84(1.94–4.15)***	0.57(0.09–3.63)
20–29	1.64(1.19–2.26)**	0.84(0.37–1.91)
30–39	1.10(0.75–1.62)	0.25(0.05–1.29)
40–49 (ref.)	1.00	1.00
**Place of residence**		
Rural	1.60(1.18–2.17)**	1.39(0.99–1.96)
Urban (ref.)	1.00	1.00
**Region of residence**		
Hhohho	1.52(1.09–2.13)*	0.78(0.57–1.07)
Shiselweni	1.29(0.97–1.72)	1.05(0.80–1.38)
Lubombo	1.37(1.03–1.82)*	1.12(0.71–1.79)
Manzini (ref.)	1.00	1.00
**Community socioeconomic disadvantage**		
High	1.50(1.10–2.03)**	1.44(1.03–2.00)*
Low (ref.)	1.00	1.00
N	4,686	4,761

***P<0.001,

**P<0.01,

*P<0.05,

AOR: adjusted odds ratio, ref.: reference, N: sample observations

**Table 4:** Model estimates from the two-level logistic regression model for factors associated with attitudes of acceptance of intimate partner violence by women aged 15–49 in Eswatini, 2010–2014.

Random effects	Empty model	Individual	Community	Final (individual + community)
MICS 2010	Model 1	Model 2	Model 3	Model 4
Community variance	0.75	0.56	0.34	0.41
Log-likelihood	-2977.22	-2766.8	-2903.49	-2737.81
ICC(%)	18.6	14.5	9.3	11.2
PCV(%)	Reference	22.3	50	40.1
AIC	5958.44	5557.59	5820.98	5509.62
Wald Chi-square		302.92***	132.66***	339.97***
MOR	2.29	2.04	1.74	1.85
N	4,686	4,686	4,686	4,686
**MICS 2014**	**Model 1**	**Model 2**	**Model 3**	**Model 4**
Community variance	0.45	0.38	0.33	0.37
Log-likelihood	-3653.87	-3272.53	-3616.56	-3256.65
ICC(%)	12.1	10.4	9	10
PCV(%)	Reference	13.9	25.4	17
AIC	7311.73	6569.06	7247.12	6547.29
Wald Chi-square		159.20***	50.49***	211.50***
MOR	1.9	1.8	1.72	1.78
N	4,761	4,761	4,761	4,761

***P<0.001,

**P<0.01,

*P<0.05,

ICC: intra-cluster correlation coefficient, PVC-proportion change in variance, MOR: median odds ratio, ref.: reference, N: sample observations

In 2010, women who were unmarried/single (AOR = 1.44, 95% CI: 1.09, 1.90), formerly married (AOR = 1.55, 95% CI: 1.16, 2.07), or women in polygynous unions (AOR = 1.78, 95% CI: 1.16, 2.74) were more likely than women in monogamous relationships to have supportive attitudes towards intimate partner violence. Only unmarried women had higher odds of accepting intimate partner violence in both the 2010 and 2014 survey years (AOR = 1.44, 95% CI: 1.09, 1.90 and AOR = 3.2, 95% CI: 1.00, 10.32; respectively).

In 2010, younger women aged 15–19, and 20–29 were more likely to accept intimate partner violence (AOR = 2.84, 95% CI: 1.94, 4.15, and AOR = 1.64, 95% CI: 1.19, 2.26; respectively) compared to those aged 40–49 years. However, in 2014, age was not associated with justifying intimate partner violence. In 2010, women living in rural areas were more likely to accept intimate partner violence (AOR = 1.60, 95% CI: 1.18, 2.17) compared to their urban counterparts. In 2014, there was no significant association between place of residence and women’s acceptance of intimate partner violence. Concerning region of residence, women from the Hhohho and Lubombo regions had more supportive attitudes toward intimate partner violence (AOR = 1.52, 95% CI: 1.09, 2.13, and AOR = 1.37, 95% CI: 1.03, 1.82; respectively) compared to women from the Manzini region in 2010. However, in 2014 there was no association between region and women’s acceptance of intimate partner violence.

In both survey years, community socioeconomic disadvantage was significantly associated with women’s acceptance of intimate partner violence. In 2010, women from communities with a high socioeconomic disadvantage had higher odds of accepting intimate partner violence (AOR = 1.50, 95% CI: 1.10, 2.03) compared to those from communities with a low socioeconomic disadvantage. Similarly, in 2014 acceptance regarding intimate partner violence was higher among women from communities with high socioeconomic disadvantage (AOR = 1.44, 95% CI: 1.03, 2.00).

As indicated in Table 4, measures of variation—between-area (community) variance, and the intra-community correlation coefficient (ICC)—depict that there was a significant variation in women’s acceptance of intimate partner violence across communities in all the fitted multilevel models. In model 1, the ICC showed that about 18.62% in 2010 and 12.05% in 2014 of the individual variance in attitudes toward accepting intimate partner violence was related to the community level and might be attributable to community factors. After adjusting for explanatory factors in models 2, 3, and 4, the community variance of women’s acceptance of intimate partner violence significantly decreased in both survey years. Likewise, the median odds ratios (MOR) values were above 1 in all the models 1 to 4 for both survey years which indicated that the communities, as defined by clusters, do appear to capture a relevant context for understanding individual women’s likelihood of justifying intimate partner violence.

The proportional change in variance (PCV) relative to model 1, for models 2, 3, and 4 shows how much of the between-community variance is explained by the additional explanatory variables. The PCV showed that 22.31% in 2010 and 13.89% in 2014 of the total variance in intimate partner variance was explained by individual-level factors. Likewise, in model 3, after controlling for only the community-level factors, the variance in intimate partner violence significantly decreased. The PCV in model 3 showed that in 2010, 50.04%, and 25.37% in 2014 of the total variance in the acceptance of intimate partner violence could be explained by community-level factors. After controlling for both the individual- and community-level factors (model 4), the variation in the acceptance of intimate partner violence across communities was also reduced. There was a PCV of 40.07% in 2010 and 16.96% in 2014, indicating that a fraction of the variance in attitudes supportive of intimate partner violence among women was explained by both the individual and community level factors. Detailed findings for 2010 and 2014 multilevel analysis can be found in the S1 File.

## Discussion

The current study compared the levels of acceptability of intimate partner violence between 2010 and 2014. The study reveals that the overall prevalence of acceptance of intimate partner violence decreased by 27.9% (from 27.6% in 2010 to 19.9% in 2014). This was also a drop from 37.8% estimated for the 2007 survey in Eswatini [14]. Further, it should be noted that the levels of acceptability of intimate partner violence in both the 2010 and 2014 surveys are lower than the average of 41% found in a study of 49 low- and middle-income countries [16]. Additionally in South Asia, the levels of acceptance ranged from 44% to 94% in Pakistan Punjab, and Afghanistan, respectively [4].

The current study also noted a reduction in the prevalence of acceptance of domestic violence for all the questions (goes out without telling partner, neglects the children, argues with him, burns food, and refuses sexual intercourse). There is limited research on the reasons for the decline or change in the attitudes toward intimate partner violence in Eswatini. Brear and Bessarab [50] argue that traditionally men yielded control over women who were seen as minors and expected to be submissive. However, this social acceptance has been waning as women’s roles seem to have been changing through gaining economic opportunities and legal protection against abuse and therefore reducing men’s power in a relationship [50].

While in general, the study revealed declining trends in terms of the acceptability of intimate partner violence in Eswatini, the proportions of women who still accept intimate partner violence are significant. This suggests that more effort in terms of information, education, and communication about intimate partner violence is still needed. There is a need to scale awareness campaigns to educate women about their rights and the overall effects of intimate partner violence. One of the policies that has been put to mitigate the problem of intimate partner violence by the government is the Eswatini Sexual Offences and Domestic Violence Act in 2018 [38].

The main objective of the study was to examine the predictors of the acceptability of intimate partner violence in Eswatini between 2010 and 2014. As expected, our study showed that both wealth (resources) and education are protective against justifying intimate partner violence. While no association between wealth and acceptance of intimate partner violence was noted in 2014, in 2010, the current study corroborates findings from literature [2, 25, 26] that women from rich households were less likely to accept intimate partner violence compared to women from poor households. Since our correlational results are indicative rather than causal of the temporal relationships between the outcome and socioeconomic status, they should be looked at cautiously when policies are being formulated.

Results from multilevel modeling results in 2010 and 2014 surveys showed that women from communities with a high socioeconomic disadvantage had higher odds of women accepting intimate partner violence than women from communities with low a socioeconomic disadvantage. Women’s willingness to accept partner violence can be significantly influenced by their economic dependence [11, 12, 24]. Women’s attitudes toward intimate partner violence may be influenced by norms, values, and practices that are sustained by systems that subjugate women in legal, social, and political institutions [27, 31]. In patriarchal societies [4], as is the case in Eswatini [30] weak civil rights for women, limited opportunities for women, and gender inequality are potential risk factors for justification of intimate partner violence. Accordingly, sustained economic empowerment of women is required through the implementation of various political, social, and legal measures, including the provision of formal and informal employment in line with the SDGs.

The current study also noted that education was negatively associated with the acceptability of intimate partner violence. Holding other factors constant in the final model, the study results showed that women with primary or less education were significantly more likely to accept intimate partner violence than women with secondary or higher education, in both the 2010 and 2014 survey years. The findings of the current study confirm literature observations [12, 16, 25] that a lower level of education was positively associated with justifying intimate partner violence while higher levels of female literacy were more likely to be the strongest protective factor for women’s societal acceptance of intimate partner violence. This finding underscores the crucial role that education plays in the emancipation of women as well as the need for the Eswatini government to continue increasing educational support and promoting women’s tertiary education to change women’s views about intimate partner violence.

The variable marital structure was associated with supportive attitudes toward intimate partner violence. Due to the likelihood that they would be sharing scarce resources, we anticipated people who were married in polygynous partnerships to be more accepting of intimate partner abuse, but this finding was not significant in the study. In both 2010 and 2014, the study found out women who had never married were more likely to have these supportive attitudes compared to those women married in monogamous relationships. This contradicts findings that found those who never partnered were less likely to accept women’s abuse by their partners [26] since marital dependency reinforces women to justify violence, especially among females with limited resources [11], and where relatives have a major role in family decisions [27]. Married women tend to tolerate intimate partner violence in circumstances where they disobey their partners or violate expected domestic duties under norms that consider them minors and entreat men to be dominant in intimate relationships [27, 29]. The circumstances in which unmarried are more accepting of intimate partner abuse require further inquiry.

The study also reveals that in 2010, but not in 2014, age was a predictor of the acceptance of intimate partner violence. Unexpectedly, young women (aged 15–29 years) were more likely to accept intimate partner violence than older women (aged 45–49 years). This result corroborates findings from other studies [2, 26]. While not conclusive in this study, earlier research has demonstrated that exposure to violence during childhood and adolescence generates norms that may lead to the acceptance of violent behaviors [20, 51]. Younger age could be linked to acceptance of intimate partner violence since it may indicate a lack of education or experience [11]. However, younger individuals are expected to be more exposed or informed because of improvements in education and legislative policies over time than older women, who may still adhere to traditional norms that normalize attitudes supportive of violence [24]. This implies that adolescent women may have a high economic dependency on intimate partners. Such a situation may not only expose them to the different forms of intimate partner violence, but also a myriad of reproductive health issues, including HIV, early marriages, pregnancies, and early childbearing. Teenage girls should therefore be a primary target audience for programming that raises awareness of intimate relationship violence since they may have minimal bargaining power or fear retaliation from rejecting masculinity norms.

While in 2014, the current study found no significant association between place of residence and women’s acceptance of intimate partner violence, in 2010, women residing in rural areas were more likely to accept intimate partner violence compared to those living in urban areas. This finding is similar to other findings [2, 25–28]. In Eswatini, traditional beliefs and practices are much valued and sustained in rural areas, especially through the hegemony of Chiefs and other traditional leaders [31, 40]. Therefore, women who were raised in rural may have experiences and views that normalize the acceptance of partner abuse of women [27, 52]. One study in Eswatini found that 52% of women did not report sexual assault because they socialized as children to accept forced sex from a partner as normal [32]. Self-blaming, stereotyping of abused women, and women embracing traditional gender roles may nurture women’s abuse by their partners [29, 32]. In Eswatini, the region of residence was an important predictor of women’s justification of intimate partner violence in the 2010 survey but not later in the 2014 survey. A region may proxy a wider community or cultural influence of shaping attitudes toward intimate partner violence [29]. The non-significance of the influence of region, and area of residence over time, probably could explain a decline in norms fostering acceptance of intimate partner violence against women as modernization and urbanization processes progress in Eswatini. The results are not conclusive as more in-depth research is needed on the underlying causes of those disadvantaged being accepting of intimate partner violence.

Our results are consistent with the social-ecological framework [3] that finds women’s individual justifying attitudes toward intimate partner violence have a community influence (i.e., ICC = 18.6%, MOR = 2.29 in 2010 and ICC = 12.1%, MOR = 1.90 in 2014). In both survey years in the multilevel models (Model 1 to 4), the MOR values were above 1 and close to 2 suggesting that community variation in the attitudes justifying intimate partner violence in Eswatini. The current study also notes from model 2 that the proportional change in variance (PCV) showed that 22.31% in 2010 and 13.89% of the total variance in acceptance of intimate partner variance was explained by the individual-level factors. The PCV in model 3 also showed that in 2010, 50.04%, and 25.37% in 2014 the total variance in the acceptance of intimate partner violence could be explained by community-level factors. Our results from the final model (model 4) showed a PCV of 40.07% in 2010 and 16.96% in 2014 indicating that a fraction of the variance of the acceptance of intimate partner violence among women was explained by both the individual and community level factors. This suggests that a comprehensive strategy to reduce intimate partner violence in Eswatini should consider the structural, community, and individual socioeconomic position of women.

### Strengths and weaknesses

The study findings were based on MICS surveys, which are nationally representative cross-sectional surveys. Since the MICS surveys are based on a robust survey sampling design and methodology, results are comparable and generalizable [4]. The study used two time points to ensure the comparison of variables across time. The study incorporates both the individual (measures of association) and community (measures of variance) analytical approaches using multilevel regression models for a better understanding of women’s acceptance of intimate partner violence [49].

The study is not without its limitations. The cross-sectional design for MICS surveys does not allow causal relationships to be identified. Due to limited variables available in MICS surveys, the severity of violence justification attitudes was not measured, and other independent variables such as women’s labor force participation, empathy, and prosocial behavior characteristics. Views on attitudes toward intimate partner violence against women were only assessed although views of men and opinions on same-sex relations on violence-supportive attitudes may be important. Last but not least, bias could have occurred due to self-reported attitude responses which are affected by social desirability, or due to other broad factors such as peer networks, and parental influence which were not explored. However, the study findings closed the gap in understanding how various social and economic characteristics may influence attitudes toward intimate partner violence in Eswatini. Further research using different approaches such as ethnographic and longitudinal studies may detail nuances of attitudes as well as give a lifecycle approach to understanding changes in attitudes.

## Conclusions

Overall, the acceptance of intimate partner violence against women is low in Eswatini. From 2010 to 2014, intimate partner violence became less acceptable. Although this is commendable, concomitant to SGDs of bringing peace, reducing inequalities, and promoting gender equality efforts and targeted interventions may help in shaping attitudes on intimate partner violence. Women who are more at risk of accepting intimate partner violence are those uneducated or with primary education. Concerning household wealth, women with few resources were more prone to justify intimate partner violence in the 2010 survey. This finding affirming resource theory, however, was not reconciled in the 2014 survey as no relation was found. Our study findings revealed the women’s differences in attitudes toward intimate partner violence were related to the community level and attributable to community factors. Women from communities with high community disadvantage are more prone to justifying intimate partner violence—in support of both the resource and social-ecological theories. Our study shows that women’s socioeconomic status disadvantage both at the individual- and community-level is associated with attitudinal acceptance of intimate partner violence. To advance gender equality in Eswatini while not excluding men, interventions, and efforts to lessen supportive attitudes toward intimate partner violence must still be promoted both at the individual and community level.

## Supporting information

S1 File Supplementary tables. (DOC)
