# Supplementary material for: Women’s socioeconomic status and attitudes toward intimate partner violence in Eswatini: A multilevel analysis
Source: PLoS One. 2023 Nov 13;18(11):e0294160. doi: 10.1371/journal.pone.0294160 (PMC10642784; doi:10.1371/journal.pone.0294160)
Supplement: S1 File — (DOC) [file pone.0294160.s001.doc]

**Supplementary Tables**

**Supplementary Table A: Adjusted odds ratios from multilevel logistic regression of attitudes of acceptance of intimate partner violence by women aged 15-49 in Eswatini, MICS**2010

|  | **Model 1** | **Model 2** | **Model 3** | **Model 4** |
| --- | --- | --- | --- | --- |
| **Fixed effects** | **AOR(95% CI)** | **AOR(95% CI)** | **AOR(95% CI)** | **AOR(95% CI)** |
| **Household wealth** |  |  |  |  |
| Poor |  | 2.19(1.70-2.82)*** |  | 1.75(1.33-2.29)*** |
| Middle |  | 1.62(1.25-2.09)*** |  | 1.40(1.07-1.83)* |
| Rich (ref.) |  | 1.00 |  | 1.00 |
| **Education** |  |  |  |  |
| None |  | 2.45(1.91-3.13)*** |  | 2.34(1.83-2.97)*** |
| Primary |  | 1.51(1.18-1.94)*** |  | 1.48(1.16-1.89)** |
| Secondary or higher (ref.) |  | 1.00 |  | 1.00 |
| **Marital structure** |  |  |  |  |
| Unmarried |  | 1.41(1.07-1.85)* |  | 1.44(1.09-1.90)** |
| Formerly married |  | 1.44(1.08-1.92)* |  | 1.55(1.16-2.07)** |
| Polygynous |  | 1.85(1.19-2.86)** |  | 1.78(1.16-2.74)** |
| Monogamous (ref.) |  | 1.00 |  | 1.00 |
| **Age group** |  |  |  |  |
| 15-19 |  | 2.91(1.99-4.25)*** |  | 2.84(1.94-4.15)*** |
| 20-29 |  | 1.62(1.18-2.23)** |  | 1.64(1.19-2.26)** |
| 30-39 |  | 1.09(0.74-1.59) |  | 1.10(0.75-1.62) |
| 40-49 (ref.) |  | 1.00 |  | 1.00 |
| **Place of residence** |  |  |  |  |
| Rural |  |  | 1.86(1.40-2.47)*** | 1.60(1.18-2.17)** |
| Urban (ref.) |  |  | 1.00 | 1.00 |
| **Region of residence** |  |  |  |  |
| Hhohho |  |  | 1.42(1.04-1.95)* | 1.52(1.09-2.13)* |
| Shiselweni |  |  | 1.40(1.07-1.84)* | 1.29(0.97-1.72) |
| Lubombo |  |  | 1.46(1.12-1.91)** | 1.37(1.03-1.82)* |
| Manzini (ref.) |  |  | 1.00 | 1.00 |
| **Community**  **socioeconomic**  **disadvantage** |  |  |  |  |
| High |  |  | 2.12(1.61-2.79)*** | 1.50(1.10-2.03)** |
| Low (ref.) |  |  | 1.00 | 1.00 |
| **Random effects** | **Empty** | **Individual** | **Community** | **Final** |
| Variance (SE) | 0.75(0.13) | 0.56(0.12) | 0.34(0.08) | 0.41(0.10) |
| Log-likelihood | -2977.22 | -2766.80 | -2903.49 | -2737.81 |
| ICC(%) | 18.6 | 14.5 | 9.3 | 11.2 |
| PCV(%) | Reference | 22.3 | 50.0 | 40.1 |
| AIC | 5958.44 | 5557.59 | 5820.98 | 5509.62 |
| Wald Chi-square |  | 302.92*** | 132.66*** | 339.97*** |
| MOR | 2.29 | 2.04 | 1.74 | 1.85 |
| N | 4,686 | 4,686 | 4,686 | 4,686 |

***P<0.001, **P<0.01, *P<0.05,

AOR: adjusted odds ratio, SE: standard error, ICC: intra-cluster correlation coefficient, PVC-proportion change in variance, MOR: median odds ratio, ref.: reference, N: sample observations

**Supplementary Table B: Adjusted odds ratios from multilevel logistic regression of attitudes of acceptance of intimate partner violence by women aged 15-49 in Eswatini, MICS2014**

|  | **Model 1** | **Model 2** | **Model 3** | **Model 4** |
| --- | --- | --- | --- | --- |
| **Fixed effects** | **AOR(95% CI)** | **AOR(95% CI)** | **AOR(95% CI)** | **AOR(95% CI)** |
| **Household wealth** |  |  |  |  |
| Poor |  | 1.49(1.05-2.11) |  | 1.12(0.76-1.65) |
| Middle |  | 1.35(0.88-2.07) |  | 1.11(0.70-1.76) |
| Rich (ref.) |  | 1.00 |  | 1.00 |
| **Education** |  |  |  |  |
| None |  | 3.71(1.61-8.52)** |  | 3.66(1.57-8.53)** |
| Primary |  | 3.44(1.30-9.09)* |  | 3.42(1.29-9.05)* |
| Secondary or higher (ref.) |  | 1.00 |  | 1.00 |
| **Marital structure** |  |  |  |  |
| Unmarried |  | 3.12(0.97-10.01) |  | 3.21(1.00-10.32)* |
| Formerly married |  | 2.59(0.85-7.89) |  | 2.70(0.89-8.17) |
| Polygynous |  | 1.28(0.71-2.33) |  | 1.23(0.68-2.22) |
| Monogamous (ref.) |  | 1.00 |  | 1.00 |
| **Age group** |  |  |  |  |
| 15-19 |  | 0.59(0.09-3.74) |  | 0.57(0.09-3.63) |
| 20-29 |  | 0.83(0.36-1.89) |  | 0.84(0.37-1.91) |
| 30-39 |  | 0.24(0.05-1.29) |  | 0.25(0.05-1.29) |
| 40-49 (ref.) |  | 1.00 |  | 1.00 |
| **Place of residence** |  |  |  |  |
| Rural |  |  | 1.44(0.94-2.21) | 1.39(0.99-1.96) |
| Urban (ref.) |  |  | 1.00 | 1.00 |
| **Region of residence** |  |  |  |  |
| Hhohho |  |  | 0.74(0.53-1.03) | 0.78(0.57-1.07) |
| Shiselweni |  |  | 1.01(0.76-1.33) | 1.05(0.80-1.38) |
| Lubombo |  |  | 1.04(0.66-1.63) | 1.12(0.71-1.79) |
| Manzini (ref.) |  |  | 1.00 | 1.00 |
| **Community socioeconomic disadvantage** |  |  |  |  |
| High |  |  | 1.74(1.36-2.23)*** | 1.44(1.03-2.00)* |
| Low (ref.) |  |  | 1.00 | 1.00 |
| **Random effects** | **Empty** | **Individual** | **Community** | **Final** |
| Variance (SE) | 0.45(0.10) | 0.38(0.15) | 0.33(0.07) | 0.37(0.13) |
| Log-likelihood | -3653.87 | -3272.53 | -3616.56 | -3256.65 |
| ICC(%) | 12.1 | 10.4 | 9.0 | 10.0 |
| PCV(%) | Reference | 13.9 | 25.4 | 17.0 |
| AIC | 7311.73 | 6569.06 | 7247.12 | 6547.29 |
| MOR | 1.90 | 1.80 | 1.72 | 1.78 |
| Wald Chi-square |  | 159.20*** | 50.49*** | 211.50*** |
| N | 4,761 | 4,761 | 4,761 | 4,761 |

***P<0.001, **P<0.01, *P<0.05,

AOR: adjusted odds ratio, SE: standard error, ICC: intra-cluster correlation coefficient, PVC-proportion change in variance, MOR: median odds ratio, ref.: reference, N: sample observations
